# Cyborg-swarm cooperation and game via affective-based brain–machine interface

**DOI:** 10.1093/nsr/nwag313

**Published:** 2026-05-28

**Authors:** Zirui Chen, Lin Zhang, Guiyong Chen, Hongru Liu, Zhikun Wang, Xinhe Zhao, Shiliang Guo, Tianming Zhao, Mingze Sun, Wenfeng Liang, Ling Qin, Mingjun Zhang, Lianqing Liu, Wenxue Wang

**Affiliations:** WINDY Lab, Department of Artificial Intelligence, Westlake University, Hangzhou 310030, China; The State Key Laboratory of Robotics and Intelligent Systems, Shenyang Institute of Automation, Chinese Academy of Sciences, Shenyang 110016, China; School of Information Science and Engineering, The Shenyang University of Technology, Shenyang 110870, China; The State Key Laboratory of Robotics and Intelligent Systems, Shenyang Institute of Automation, Chinese Academy of Sciences, Shenyang 110016, China; School of Mechanical Engineering, Shenyang Jianzhu University, Shenyang 110168, China; School of Biomedical Engineering, Tsinghua University, Beijing 100084, China; WINDY Lab, Department of Artificial Intelligence, Westlake University, Hangzhou 310030, China; The State Key Laboratory of Robotics and Intelligent Systems, Shenyang Institute of Automation, Chinese Academy of Sciences, Shenyang 110016, China; Software College, Northeastern University, Shenyang 110169, China; WINDY Lab, Department of Artificial Intelligence, Westlake University, Hangzhou 310030, China; The State Key Laboratory of Robotics and Intelligent Systems, Shenyang Institute of Automation, Chinese Academy of Sciences, Shenyang 110016, China; The State Key Laboratory of Robotics and Intelligent Systems, Shenyang Institute of Automation, Chinese Academy of Sciences, Shenyang 110016, China; School of Mechanical Engineering, Shenyang Jianzhu University, Shenyang 110168, China; Laboratory of Hearing Research, School of Life Sciences, China Medical University, Shenyang 110122, China; School of Biomedical Engineering, Tsinghua University, Beijing 100084, China; The State Key Laboratory of Robotics and Intelligent Systems, Shenyang Institute of Automation, Chinese Academy of Sciences, Shenyang 110016, China; The State Key Laboratory of Robotics and Intelligent Systems, Shenyang Institute of Automation, Chinese Academy of Sciences, Shenyang 110016, China

**Keywords:** cyborg swarm, brain–machine interface, affective state, multi-agent reinforcement learning, bio-hybrid robotics

## Abstract

The integration of biological organisms with robotic systems has enabled hybrid cyborg platforms that combine biological sensory agility with electromechanical precision. However, existing cyborg systems predominantly rely on unidirectional stimulus-driven control, treating animals as bio-actuators while neglecting their intrinsic cognitive states. To bridge this gap, we present a closed-loop cyborg-swarm architecture that utilizes the animal’s internal affective state (fear) as a high-level trigger to modulate robotic swarm strategies. Specifically, we developed a lightweight, real-time wireless brain–machine interface (BMI) to record local field potentials from the mouse basolateral amygdala. To ensure robust decoding in freely moving subjects, we implemented a dual-threshold detection algorithm that identifies fear states based on elevated $\beta$-band power (15–30 Hz) and suppressed high-frequency noise, effectively rejecting motion artifacts. This decoded intent drives a dual-mode control framework: under baseline conditions, the system operates in a proportional-integral-derivative (PID)-based Exploration Mode; upon detection of fear, it autonomously switches to an Interaction Mode governed by Multi-Agent Deep Deterministic Policy Gradient. In this mode, a heterogeneous robotic swarm (comprising a MouseBot and an ally micro aerial vehicle (MAV)) executes coordinated adversarial defense strategies against an enemy MAV. Experimental results in a search-interference game demonstrate that biological affective signals can successfully trigger millisecond-level control authority switching, enabling the emergence of complex bio-machine cooperative behaviors. This work marks a paradigm shift from physical-level interaction to cognitive-level bio-hybrid cooperation, validating a scalable framework for emotion-modulated cyborg swarms.

## Teaser

A mouse affective neural signal triggers robotic swarm strategies for adaptive bio-hybrid cooperation in adversarial games.

## INTRODUCTION

The rapid advancement of neural interface technologies has accelerated the deep integration of robotic systems with biological organisms, giving rise to hybrid bio-machine systems known as cyborgs [1–3]. By fusing biological sensory-motor agility with electromechanical precision, these systems offer new paradigms for next-generation intelligence that leverage the complementary strengths of both worlds.

A dominant paradigm in cyborg research involves stimulus-driven control, where machines modulate animal behavior through external stimuli. Foundational studies have realized the guidance of beetles, honeybees, pigeons, or cockroaches via neuromuscular or brain stimulation [4–9]. Essentially, these systems function as ‘bio-actuators’, establishing a robust unidirectional control channel from machine to organism. Recent advancements have further optimized this execution layer, employing reinforcement learning to assist navigation [10] or utilizing ultra-thin interfaces to stabilize locomotion [11,12]. However, despite these improvements in actuation precision, the control loop predominantly relies on external behavioral measurements (for example, location tracking), capturing only the result of the animal’s action rather than its internal cognitive state.

To achieve true hybrid intelligence, the system must bridge the gap between ‘machine execution’ and ‘biological intent’. This necessitates the integration of brain–machine interfaces (BMIs) not merely as stimulation tools, but as state observers that inject biological internal intent, such as affective states or motion intentions, directly into the control loop [13,14]. Unlike external observation, direct neural decoding provides a multi-channel, low-latency window into the biological agent’s intrinsic assessment of the environment. While active BMIs have been successfully applied to motor decoding for neuroprosthetics and teleoperation [8,15–19], and deep learning has enabled accurate decoding of specific intentions [20,21], the integration of affective feedback (for example, fear or anxiety) as a critical state variable for system-level decision-making remains largely unexplored. Conventional emotion recognition often relies on slow, error-prone facial or behavioral analysis [22,23], and bulky wired interfaces hinder the free interaction required for dynamic tasks [24,25].

However, effectively integrating this biological intent into a cyborg swarm remains a significant challenge, particularly in the context of high-dimensional perception and decision-making. Modern purely robotic swarms have evolved to handle complex adversarial games and cooperative manipulation using multi-agent reinforcement learning (MARL) and sophisticated planning algorithms [26–32]. In contrast, current state-of-the-art cyborg swarms, such as the insect swarm demonstrated by Bai *et al*. [33], primarily rely on heuristic rules or physical reflexes for coordination in soft terrains. While effective for physical adaptation, these reactive approaches are incompatible with the requirements of high-level cognitive tasks. They lack the mechanism to map abstract biological intent (for example, fear or strategic need) into the decision layer, rendering them incapable of handling dynamic environments that require complex strategic interactions. Consequently, a critical gap exists: how to seamlessly map biological intent into a high-dimensional decision space to realize sophisticated bio-machine collaborative games.

To bridge this compatibility gap, we develop a closed-loop bio-computational framework specifically designed to address the two identified challenges: uplinking biological intent and realizing high-dimensional decision-making. Instead of a loose assembly of hardware, the system is architected as a dual-layer control loop. At the perceptual layer, we employ a lightweight wireless BMI to extract the mouse’s internal affective state (fear) as a real-time binary trigger derived from multi-channel local field potential (LFP) recordings, solving the ‘uplink’ problem by converting multi-channel biological signals into machine-readable states. At the decision layer, we implement a Multi-Agent Deep Deterministic Policy Gradient (MADDPG) framework to serve as the system’s ‘cognitive core.’ This algorithm is specifically tasked with incorporating this affective trigger into a high-dimensional robotic state space to generate coordinated strategic actions for the robotic agents (MAVs and MouseBot), thereby ensuring the decision-layer compatibility between the biological and mechanical sub-systems.

To validate the efficacy of this framework in handling high-dimensional interactions, we construct a collaborative–adversarial game task as a stress-test environment. Unlike simple navigation tasks that can be solved by heuristic rules, this scenario involving a target-seeking MouseBot team and an active adversarial micro aerial vehicle (MAV) creates a complex state space where biological safety and mission success are dynamically coupled. The game forces the system to rely on the real-time uplink of the mouse’s fear state: without this affective trigger derived from multi-channel neural recordings, the robotic team cannot anticipate the adversary’s threat level; without the MADDPG-based decision-making, the team cannot execute the precise cooperative maneuvers required to shield the mouse. Thus, this task serves not merely as a demonstration, but as a critical validation of the system’s ability to close the loop between biological intent and complex robotic strategy.

To the best of our knowledge, this work presents the first integration of intrinsic affective states into a multi-agent adversarial game framework, marking a paradigm shift from physical-level interaction to cognitive-level bio-hybrid cooperation. Central to this contribution is the establishment of a lightweight wireless BMI pipeline that robustly decodes fear-related neural signals from freely moving mice. This creates a critical ‘alert trigger’ for the robotic system, surpassing the inherent latency and ambiguity of traditional external behavioral observations. Building on this real-time affective neural trigger, we introduce a cooperative–adversarial framework driven by MADDPG. Unlike heuristic-based swarms that rely on pre-defined rules, our approach empowers the hybrid system to learn emergent strategic behaviors, such as active MAV intimidation and coordinated protection, directly from dynamic bio-machine interactions. Ultimately, by demonstrating a complete closed-loop architecture where biological affective signals modulate robotic swarm behaviors in real time, we bridge the gap between biological sensing and computational game theory, validating a scalable foundation for future cognitive cyborg swarms.

## RESULTS

The results are organized to systematically validate the construction and efficacy of the proposed closed-loop bio-computational architecture, progressing from module-level performance to system-level emergent intelligence. To realize the critical ‘uplink’ of biological intent, we first characterize the neural decoding pipeline, validating its accuracy and latency in extracting fear-related states from freely moving mice. Complementing this perceptual layer, we present the MouseBot platform performance to ensure stable physical actuation and multimode control. Building upon these hardware foundations, we evaluate the computational decision layer (MADDPG), demonstrating its convergence and ability to handle high-dimensional state inputs. Finally, we integrate these components into the collaborative–adversarial game task, providing empirical evidence of the complete closed-loop interaction where biological affect successfully modulates robotic swarm strategy in real-time.

### Affective-based BMI system for mice

Adapting the spectral biomarker methodology established by Kim *et al*. [34], we utilized the power of beta oscillations (15–30 Hz) in the basolateral amygdala (BLA) as a real-time neurophysiological biomarker for intrinsic fear states. Unlike transient gamma bursts, beta activity offers a robust signature of sustained fear, making it particularly suitable for continuous closed-loop modulation.

**Figure 1.**
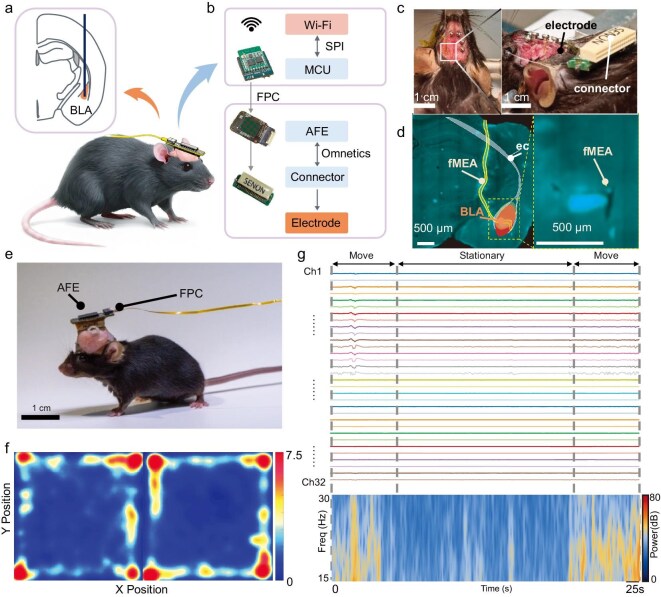
Overview of the lightweight real-time wireless BMI platform for mice. (a) Mouse brain atlas. The red region indicates the BLA, and the black lines illustrate the implanted flexible electrode sites. (b) Exploded view of the BMI system. The head-mounted module includes the electrode interface and 32-channel analog front-end (AFE), while the Wi-Fi wireless communication module and power supply are installed on the vehicle. SPI: serial peripheral interface; MCU: microcontroller unit. The two modules are connected via flexible printed circuits (FPCs). (c) Electrode implantation. Left: stereotaxic targeting of electrode implantation sites. Right: schematic of the electrode implantation structure. (d) Postmortem mouse brain slice. The yellow outline shows the electrode implantation trajectory; right: magnified view of the flexible electrode tip in the BLA. fMEA: flexible microelectrode array; ec: external capsule. (e) Photograph of a mouse freely moving while wearing the BMI system. Results indicate that the head-mounted system does not interfere with natural locomotion. (f) Free-moving trajectory of the mouse within the experimental arena. Movement patterns of BMI-equipped mice show no significant difference compared to control mice. (g) Recorded 32-channel neural signals during free movement. Bottom: corresponding LFP time–frequency spectrograms.

We developed a lightweight, real-time wireless BMI system for small rodents to acquire emotion-related neural signals from freely moving mice. The system was designed for the BLA, a key brain region for emotional regulation [35,36] (Fig. 1a), and uses 32-channel high-density flexible electrodes to monitor neural activity in real time. The overall system adopts a modular architecture [37]: the head-mounted component includes the implanted electrodes, interface, and analog front-end, while the onboard wireless communication module and power supply are installed on a robotic vehicle (Fig. 1b). Electrode implantation was guided by stereotaxic techniques (Fig. 1c, Methods), and postmortem brain slice staining confirmed that the recording sites aligned with the target BLA region (red circles in Fig. 1d).

Thanks to the modular and separated system design, the head-mounted portion weighs only 1.439 g, allowing natural mouse behavior without impairment (Fig. 1e). Open-field tracking analysis (Fig. 1f) confirmed that locomotion metrics—including distance traveled, speed, and exploration area—were not significantly different from normal mice. The system stably records 32-channel electrophysiological signals in freely moving mice (Fig. 1g), providing high spatiotemporal resolution for studying the neural encoding of emotional behaviors.

**Figure 2.**
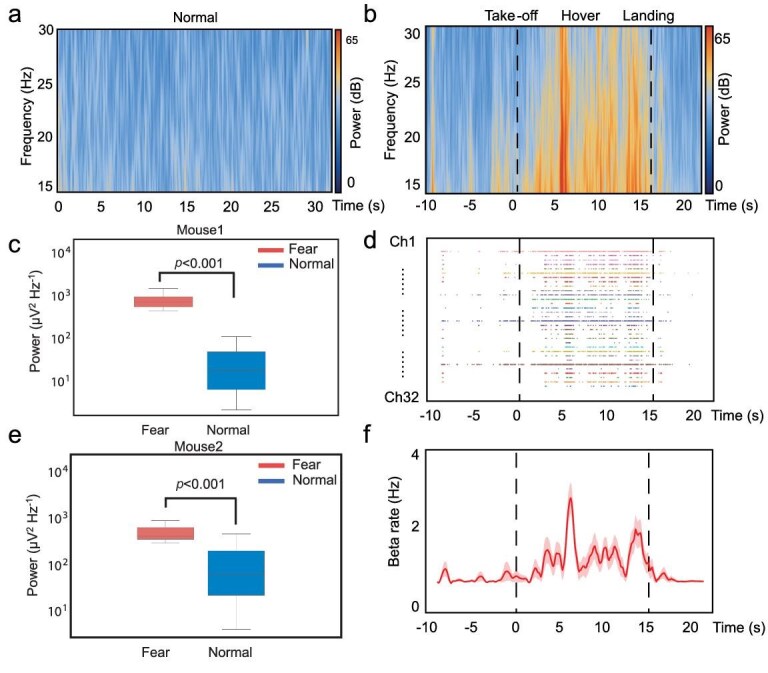
Functional analysis of $\beta$-band neural oscillations during fear. (a, b) Time–frequency spectrograms of average channels in a mouse during resting (a) and fear (b) states. Dashed lines indicate MAV takeoff (0 s) and landing (15 s). (c, e) Comparison of average $\beta$-band power between resting and fear states for mice 1 (c) and 2 (e), showing significant increases during fear. (d) Raster plot illustrating the temporal density of bursts under fear. (f) Burst rate analysis across LFP channels, showing increased $\beta$-band burst rates following MAV takeoff and return to baseline after landing.

To characterize fear-related neural signatures induced by MAV exposure and support the design of the real-time decoding algorithm, we first conducted experiments where an operator manually controlled MAV takeoff and hovering while recording BLA neural dynamics. Time-frequency analysis using Morlet wavelets was performed on multi-channel LFPs in the BLA during resting (Fig. 2a) and fear states (Fig. 2b). We observed a marked increase in $\beta$-band (15–30 Hz) neural activity from MAV takeoff (time 0, Fig. 2b) to hover, consistent with previously reported findings [38]. Repeated MAV-induced fear experiments across multiple mice, analyzed using the Mann–Whitney U test, confirmed that $\beta$-band power was significantly higher in the fear state than in the resting state (Fig. 2c and e).

To quantify the robustness of these signals, we performed average firing rate analysis across multiple channels (Fig. 2d). Results indicated that $\beta$-band burst rate was significantly enhanced during fear, and burst rates across channels showed high consistency (Fig. 2d).

Together, these offline results establish $\beta$-band oscillatory power as the biomarker used by our closed-loop dual-threshold decoder.

### MouseBot interaction system and cyborg-swarm system

**Figure 3.**
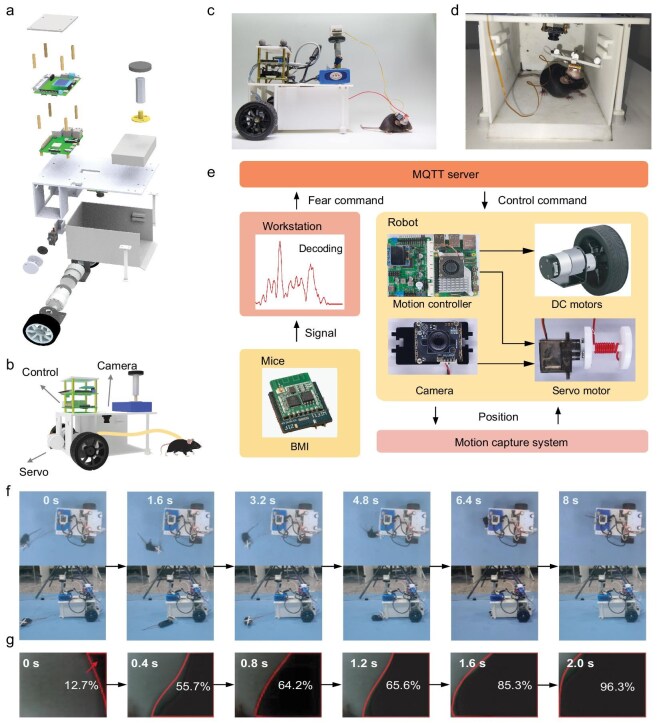
Overview and workflow of the MouseBot system. (a) Exploded view of the MouseBot. Major vehicle modules are shown, including the drive unit, control module, power system, and wireless communication unit. (b) MouseBot modeling. 3D model illustrating system structure and mouse interaction interface design. (c) Photograph of the MouseBot, showing the actual experimental vehicle and its size scale. (d) Mouse after retraction into the vehicle. Demonstrates safe recovery of the mouse inside the cabin. (e) Workflow of the MouseBot system. Illustrates the full process from mouse detection and localization to retrieval and recovery. MQTT: message queuing telemetry transport; DC: direct current. (f) Retraction process of the mouse. Shows the dynamic process of bringing the mouse back into the vehicle. (g) Mouse entering the cabin. Demonstrates autonomous entry of the mouse and system recognition and localization.

We developed a novel MouseBot collaborative system to investigate interactions between animals and robots in dynamic and adversarial tasks. The MouseBot consists of a differential-drive vehicle and a mouse equipped with a BMI (Fig. 3a and b). The mouse is tethered to the vehicle via a lightweight string: one end is attached to the mouse’s head ring, and the other is wound around a servo-driven spool at the rear of the vehicle (Fig. 3c). Under normal conditions, the vehicle passively follows the mouse without interfering with its natural behavior, allowing the animal to explore autonomously and naturally; however, when the BMI system detects fear-related neural signals, the MouseBot switches control modes. When required by the experimental task, the servo mechanism rapidly retracts the tether, smoothly drawing the mouse back into the cabin (Fig. 3d). A camera mounted inside the cabin monitors the area of black pixels in the captured images to verify whether the mouse has fully entered the vehicle (Fig. 3c). In coordination with other agents, the MouseBot can safely transport the mouse to designated target points, establishing a foundation for fully closed-loop bio-robot interaction experiments.

**Figure 4.**
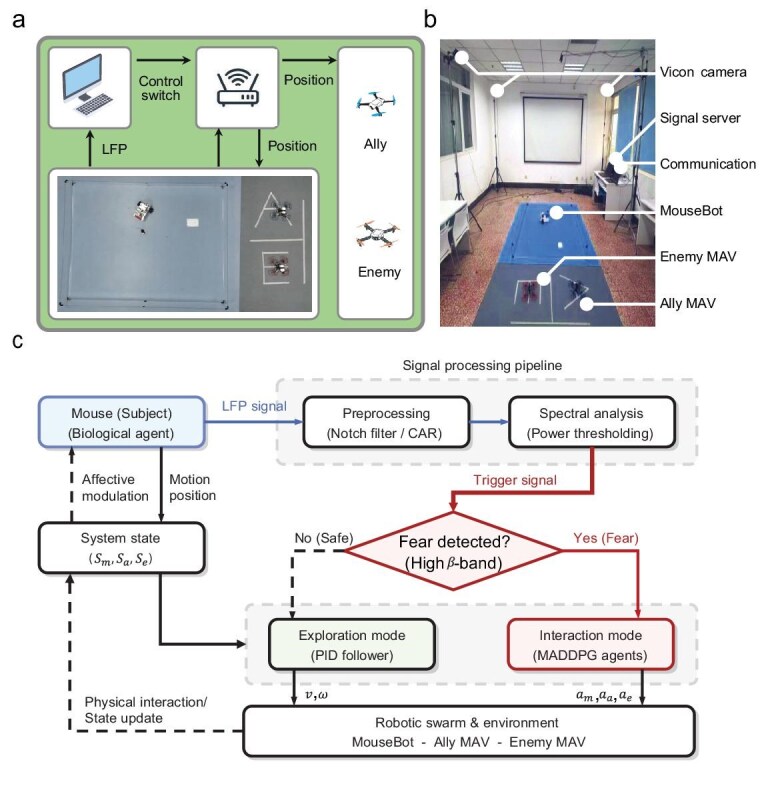
Overview of the cyborg-swarm system. (a) Conceptual illustration of the cyborg-swarm system, showing inter-agent communication, cooperation, and environmental interaction mechanisms within the hybrid collective. (b) Experimental arena layout used for cyborg-swarm validation, depicting the spatial partitioning and task zones designed to evaluate collective coordination and task performance. (c) Signal flow and dual-mode control mechanisms in the validation environment.

To enable interactive game behavior between the mouse-driven MouseBot and both ally and enemy MAVs, we developed a hybrid bio-machine collaborative–adversarial system, encompassing both hardware and software components. The overall structure is shown in Fig. 4a and b, which illustrate the system concept and physical implementation, respectively.

The cyborg-swarm system comprises six key components: the MouseBot, enemy MAV, ally MAV, signal processing server, motion capture system, communication hub, and experimental arena. Both MAVs are equipped with onboard computers and neural processing units capable of real-time inference, enabling autonomous decision-making during dynamic game interactions. The MouseBot carries a flexible BMI to continuously acquire neural signals from the mouse. These signals are transmitted to the processing server for decoding of emotional states, determining whether a control-mode switch should occur.

The signal flow and control allocation logic are illustrated in Fig. 4c. The architecture consists of two parallel control loops mediated by a bio-signal trigger. First, raw LFP signals acquired from the subject (mouse) undergo a preprocessing pipeline, including notch filtering and common average referencing (CAR), to isolate neural features. Subsequently, spectral analysis computes the real-time power in the $\beta$-band. This value serves as a decision variable: under baseline conditions (‘Safe’), the system operates in Exploration Mode, employing a proportional-integral-derivative (PID) follower to maintain physical coupling between the MouseBot and the mouse. Upon detection of a high-power $\beta$-band event (‘Fear’), the system triggers a mode switch to Interaction Mode. In this state, the control authority is transferred to the MADDPG agents, which generate coordinated joint actions ($\mathbf {a}_m, \mathbf {a}_a, \mathbf {a}_e$) based on the global system state ($S_m, S_a, S_e$) to execute adversarial defense strategies.

### Cyborg-swarm game and policy training

To design and evaluate strategies before real-world deployment, we constructed a multi-agent simulation environment that mirrors the physical setup. The task is formulated as a search–interference game involving three entities: the MouseBot and its ally MAV (search team), and an enemy MAV (interference agent). The MouseBot must locate a hidden target point *P*, while the enemy MAV blocks or intercepts its motion. The ally MAV supports the MouseBot by protecting it and driving away the enemy MAV. This environment enables high-throughput iterative learning under realistic dynamics, mitigating the high cost and risk associated with physical trials.

**Figure 5.**
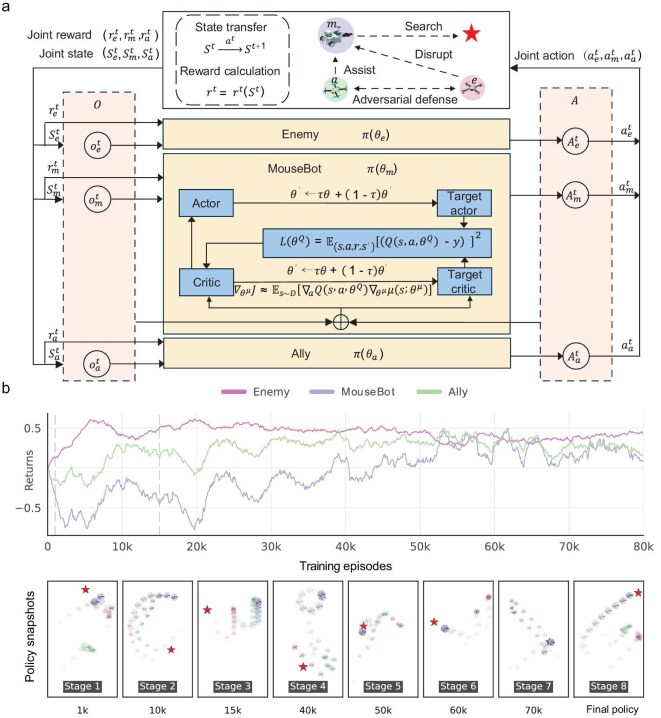
Overview of the policy training process. (a) Block diagram of the reinforcement learning framework. (b) Training return curve showing the evolution of cumulative return across iterations and policy visualization at different training stages, showing the evolution of agent behaviors from early exploration to stable performance.

We adopt the MADDPG framework [26] for training cooperative and competitive policies. Following the ‘centralized training and decentralized execution’ paradigm, all agents share global state information during training to accelerate convergence and improve robustness, while decisions during execution rely solely on local observations to ensure deployability. As shown in Fig. 5a and b, the reward trajectories exhibit multi-stage non-monotonic fluctuations, reflecting the self-balancing dynamics typical of emergent strategies in multi-agent games [39]. Notably, the ally MAV and enemy MAV display a strong negative correlation in reward trends, while the MouseBot’s performance aligns closely with its ally, indicating coupled cooperation–game behaviors.

During strategy evolution (Fig. 5b), we observed distinct phases of co-adaptation among the agents. Initially (Stage 1), all agents engaged in random exploration near the origin. Subsequently, the Enemy was the first to develop a coherent policy, learning to chase and drive the MouseBot away (Stage 2), which rapidly evolved into a sophisticated interception strategy to block the MouseBot’s path (Stage 3). In response, the Ally learned to engage the Enemy in close-quarters combat, creating opportunities for the MouseBot to approach the target amidst the chaos (Stages 4–6). Ultimately, the Ally refined its policy to actively repel the Enemy, establishing a stable escort strategy that ensured the MouseBot’s safe passage (Stages 7–8).

It is important to note that in the simulation, the MouseBot is modeled as a non-holonomic differential-drive agent whose motion policy is also learned via MADDPG. The simulation does not attempt to replicate the mouse’s internal cognitive or emotional states; rather, its purpose is to train the MAV agents’ cooperative and adversarial strategies in a high-throughput virtual environment. The genuine biological contribution—the mouse’s real-time fear assessment—enters the system exclusively in the physical closed-loop experiments, where detected BLA $\beta$-band activity serves as the trigger for control-mode switching. This separation of concerns is by design: the simulation optimizes robotic strategy, while the living animal provides the affective state observation that cannot be faithfully modeled *in silico*.

### Experimental results

**Figure 6.**
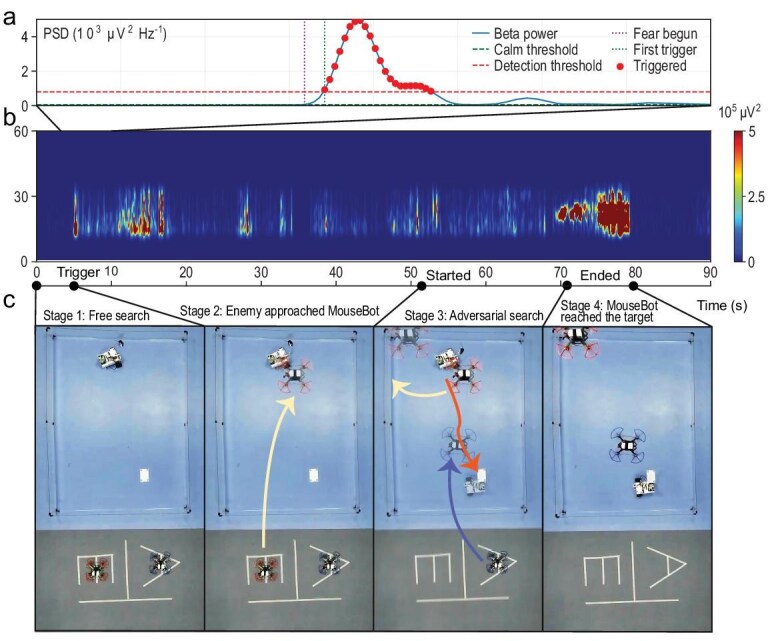
Evolution of states and control signals in a single representative trial. (a) Online detection of fear-related neural signatures, where transient oscillations and amplitude increases in the BLA signal trigger the control authority transfer. PSD: power spectral density. (b) Continuous LFP recording across the entire experimental duration, showcasing the transition from baseline to activated states. (c) Temporal alignment of the four experimental stages: free search, threat induction, adversarial search, and task termination.

Based on the integrated system described above, we conducted a series of search–interference experiments combining neural signal analysis with multi-agent game strategies. Experimental observations reveal that the mouse’s BLA activity exhibits clear temporal synchronization with the robotic system’s operational states, validating the effective coupling between neural dynamics and collective task evolution. As illustrated in Fig. 6, the experimental procedure is categorized into four distinct stages: (i) free search, (ii) threat induction (enemy MAV approach), (iii) adversarial search, and (iv) termination (target reached). Across these stages, neural signals exhibited distinct stage-specific patterns that served as decisive triggers for autonomous task switching. The evolution of the LFP signals throughout the entire experimental procedure is depicted in Fig. 6b, with the corresponding operational stages synchronized and annotated in Fig. 6c.

In the initial stage, the mouse autonomously navigated the MouseBot via the flexible BMI. During this period, $\beta$-band neural activity remained at a stable baseline with low amplitude and frequency. In the second stage, the intrusion of the enemy MAV induced an acute stress response in the mouse. This was characterized by increased LFP amplitudes and transient irregular oscillations; these fear-related neural signatures were accurately captured by our online detection algorithm (Fig. 6a).

Upon detecting these specific neural patterns, the system transitioned to the third stage, triggering a control authority transfer. Control of the MouseBot shifted from the mouse to the onboard controller, while the ally MAV intervened to execute a pre-trained adversarial strategy. This phase was marked by pronounced LFP peaks, reflecting heightened neural activation. Finally, in the fourth stage, both the MouseBot and MAVs withdrew upon task completion. The neural signals gradually receded to baseline, signifying a return to the initial resting state.

**Figure 7.**
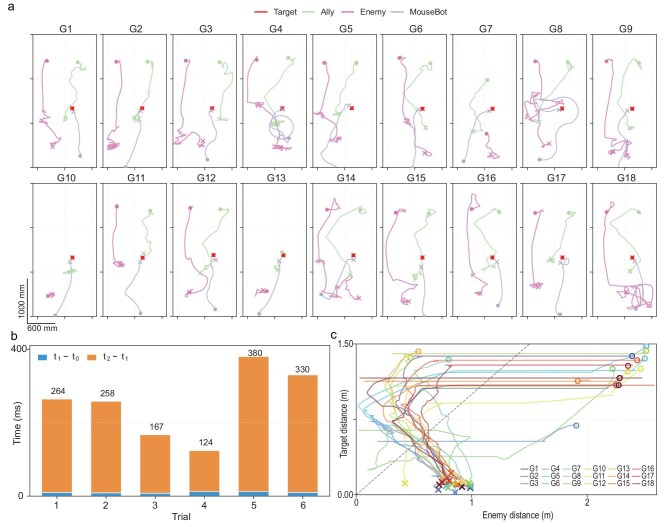
Superimposed trajectories and phase portraits across repetitive trials. (a) Overlaid spatial trajectories of 18 repeated attack–defense games, demonstrating high behavioral consistency and task success across different subjects. (b) Distribution of system latency, showing a neural signal detection time of approximately 10 ms and a total control mode switching response time of 100–400 ms. (c) Phase portrait of the relative distances between the MouseBot, the target, and the enemy, illustrating the transition from the threat-driven approach to stable convergence at the target.

To verify the robustness of the system, we conducted 18 repeated trials using two different mice. All trials successfully concluded the attack–defense game (Fig. 7a), exhibiting behavioral consistency with the representative results shown in Fig. 6. The system’s performance was further quantified through signal detection latency and response time. As shown in Fig. 7b, across recorded trials, the detection latency was approximately 10 ms, with a total system response time (from trigger to mode switch) of 100–400 ms.

Furthermore, Fig. 7c illustrates the phase portrait analyzing the spatial relationship between the MouseBot, the target, and the enemy. The trajectories reveal a two-stage dynamic: initially, the distance to the enemy decreases during the threat phase; subsequently, upon the intervention of the ally MAV and the initiation of the adversarial game, the MouseBot–enemy distance increases while the distance to the target concurrently decreases. All phase trajectories converged to a stable region, representing the successful attainment of the target while maintaining a safe clearance from the enemy. These results demonstrate a robust synchronization between neural signaling and multi-agent coordination, confirming the feasibility of the proposed closed-loop ‘neural–robotic–game’ control architecture.

## DISCUSSION

### Paradigm shift: from physical actuation to cognitive interaction

To position our work within the broader landscape of cyborg systems, we provide a comparative analysis in Table 1. Historically, the field has evolved from unidirectional ‘remote control’ [4,9] to more autonomous systems utilizing collaborative UAVs for guided navigation [40]. A significant recent leap was achieved by Bai *et al*. [33], who demonstrated a cyborg-insect swarm capable of traversing soft terrains through physical cooperation. However, as illustrated in Table 1, these state-of-the-art systems primarily operate on heuristic or reflex-based control logic. Specifically, the swarm behaviors in [33] emerge from local physical interactions (for example, pulling a trapped peer), which essentially solves a physical optimization problem but lacks a higher-level decision layer to handle abstract risks or adversarial intent.

**Table 1.** Comparison of control paradigms and functional capabilities across representative cyborg systems. This work distinguishes itself by introducing closed-loop affective feedback and high-dimensional adversarial strategies.

Study	Organism	System scale	Control & interaction logic	Bio-signal role	Task domain
Talwar *et al.* (2002) [4]	Rat	Single agent	Open-loop stimulation (behavioral conditioning)	None (unidirectional)	Waypoint navigation
Li and Zhang (2016) [9]	Cockroach	Single agent	Human-in-the-loop (SSVEP) (teleoperation)	None (unidirectional)	Path following
Zheng *et al*. (2025) [40]	Rat	Single agent	UAV-guided path planning (external sensing)	Motion/position feedback (external)	Autonomous navigation
Bai *et al*. (2025) [33]	Beetle	Homogeneous swarm	Heuristic rules & reflexes (physical coupling)	Local physical interaction	Terrain adaptation & rescue
**This work**	**Mouse**	**Heterogeneous team**	**Closed-loop MARL (MADDPG) (game-theoretic)**	**High-dim affective state (intrinsic intent trigger)**	**Adversarial defense & strategy**

In contrast, our system bridges this ‘cognitive gap’ by elevating the interaction from physical reflexes to strategic gaming. By decoding the mouse’s intrinsic fear state via BMI, we treat the biological agent not merely as a mechanical actuator, but as a high-sensitivity state observer within the control loop. The introduction of MADDPG enables the heterogeneous team to incorporate this binary affective trigger into a high-dimensional robotic state space and generate emergent cooperative strategies, such as active intimidation and formation shielding, that are unattainable through simple heuristic rules. This shift from physical coupling to informational and strategic coupling represents a fundamental step towards next-generation cyborg swarms capable of surviving and succeeding in dynamic, adversarial environments.

### Affective states as high-level control variables

This study pioneers the integration of intrinsic emotional states as pivotal control variables in bio-machine hybrid systems. While traditional cyborg control frameworks predominantly rely on overt motor signals or behavioral cues, our results show that affective neural features—specifically, $\beta$-band oscillations in the BLA—can serve as robust, real-time triggers for authority allocation. By leveraging emotional signals, future cyborg systems could move beyond simplistic ‘motion decoding’ toward complex ‘intent and state modulation’, enabling agents with heightened environmental awareness and bridging neuroscience with robotics to develop emotionally responsive intelligent systems.

### Bio-driven heterogeneous multi-agent coordination

The MouseBot platform exemplifies a novel ‘bio-driven–robot-assisted’ architecture, establishing a dynamic equilibrium between biological intuition and robotic precision. Control authority can autonomously switch between modes based on the mouse’s detected stress level, demonstrating how affective cues can orchestrate complex multi-agent cooperation. Beyond single-agent interaction, we extend these principles to heterogeneous collectives [41]. Incorporating reinforcement learning (for example, MADDPG) allows for principled coordination and game-like dynamics within the cyborg-swarm, enabling autonomous role switching through self-play. This demonstrates that computational models can effectively bridge the gap between biological perception and robotic execution, suggesting the potential for using neural signals as reward modifiers in policy adaptation.

### Limitations and future directions

Despite these advances, several limitations remain. First, current emotional decoding is restricted to binary fear-related transitions; a more nuanced spectrum of affective states remains to be explored. Second, the wireless BMI requires improvements in latency and robustness against electromagnetic interference in complex environments. Finally, the scale of the hybrid swarm is still limited. Future work should focus on developing high-dimensional neural representations for multi-emotion regulation, constructing end-to-end neuro-reinforcement learning frameworks that seamlessly unify emotion, decision-making, and action, and exploring adaptive strategy evolution in larger scale cyborg-swarms. Such efforts could enable the emergence of emotionally adaptive, cognitively integrated, and large-scale hybrid intelligent collectives applicable to ecological monitoring, disaster response, and strategic defense scenarios.

## METHODS

The 32-channel flexible electrode arrays were fabricated on 4-inch silicon wafers by photolithographic patterning of Cr/Au traces on a polyimide substrate, followed by polyimide encapsulation, ICP opening of recording sites and I/O pads, SU-8 reinforcement, release from the wafer, and ethylene oxide sterilization before assembly with the wireless neural recorder. Detailed fabrication parameters are provided in Note S1.

BLA LFPs were analyzed offline using band-pass filtering, common average referencing, Morlet time-frequency decomposition, and Mann–Whitney U tests for β-band (15–30 Hz) power comparisons. In closed-loop experiments, neural data were processed online in sliding windows, with artifact rejection, Welch PSD estimation, and a dual-threshold β/high-frequency trigger used to classify the state as Safe or Fear. Full signal-processing equations and thresholds are given in Note S2.

The MouseBot used a Raspberry Pi and STM32 controller for ROS-based pose acquisition, MQTT communication, PID following, differential-drive actuation, and servo-based mouse retrieval after fear-state detection. Vicon tracking provided real-time poses for the vehicle and the mouse, and an onboard camera verified successful retrieval by measuring the black-pixel ratio inside the cabin. Hardware and control details are provided in Note S3.

The experimental system integrated the MouseBot, ally MAV, enemy MAV, signal-processing server, motion-capture system, communication hub, and arena. MAVs supplied threat and assistance roles, while the server decoded LFPs and the communication hub distributed low-latency control commands. System engineering details are provided in Note S4.

For policy training, we extended the Multi-Agent Particle Environment to a 2-vs-1 pursuit-evasion game with heterogeneous MouseBot and MAV dynamics. MADDPG policies were trained under centralized training and decentralized execution with asymmetric rewards for target approach, collision avoidance, adversarial interaction and ally coordination. Reward functions, network architecture and hyperparameters are provided in Note S5.

## Supplementary Material

nwag313_Supplemental_File

## Data Availability

To facilitate reproducibility and open science, we have open-sourced the complete project resources. The experiment video and trajectory logs are available at GitHub: https://github.com/czr-gif/Experiment-Data-for-Cyborg-Swarm-Cooperation-and-Game-via-Affective-based-Brain-Machine-Interface.
