# Supplementary material for: Cyborg-swarm cooperation and game via affective-based brain–machine interface
Source: Natl Sci Rev. 2026 May 28;13(13):nwag313. doi: 10.1093/nsr/nwag313 (PMC13309927; doi:10.1093/nsr/nwag313)
Supplement: nwag313_Supplemental_File [file nwag313_supplemental_file.pdf]

Supplementary Data for
Cyborg-Swarm Cooperation and Game via Affective-based Brain-Machine Interface

Zirui Chen et al.

Supplementary Note 1. Fabrication of the 32-channel flexible electrodes

The 32-channel flexible electrode arrays designed for high-density neural signal recording feature contact sites of 50×80 μm with a spacing of 200 μm. The device was fabricated on 4-inch silicon wafers using conventional photolithography.

First, a 2 μm thick polyimide film (HD-2611, HD Microsystems) was spin-coated and thermally cured to serve as the substrate. A Cr/Au (10 nm/100 nm) metal layer was then patterned onto the polyimide via photolithography using AR-BR 5480 and AZ 601 photoresists. This was achieved using a manual mask aligner (URE-2000/35, Chengdu Jingpu Technology Co., Ltd) and magnetron sputtering (JCP500, Beijing Technol Science Co., Ltd), followed by a standard lift-off process.

Subsequently, a top encapsulation layer of polyimide was applied using the same parameters as the substrate. The stacked layers were patterned with AZ4620 positive photoresist (6 μm) and dry-etched using inductively coupled plasma equipment (NE-550H, ULVAC) to define the array outline and expose the recording sites and I/O pads.

To provide structural reinforcement, SU-8 2050 was spin-coated at 3000 rpm. Following soft baking, exposure, post-exposure bake and development, reinforcement structures with a height of approximately 50 μm were formed. Finally, the completed electrode arrays were released from the silicon substrate using water-soluble tape (Water-soluble wave solder 5414, 3M). The arrays were immersed in deionized water overnight at room temperature to fully dissolve the tape. Prior to assembly with the wireless neural recorder, the arrays were sterilized in an ethylene oxide sterilization system (HM78, Tianjin SATOU Environmental Machinery Co., Ltd).

Supplementary Note 2. Neural signal analysis and real-time decoding

Neural signals from the basolateral amygdala were recorded via a multi-channel implantable brain-machine interface. For offline visualization and statistical analysis, signals were preprocessed using standard band-pass filtering and common average referencing:

$$\hat{x}_i(t) = x_i(t) - \frac{1}{N}\sum_{j=1}^{N} x_j(t), \tag{1}$$

where $x_i(t)$ is the raw signal from channel i and N is the total number of channels. Time-frequency analysis was performed using Morlet wavelet convolution to characterize neural oscillatory dynamics with high temporal resolution. Power was expressed in decibels:

$$P_{dB}(t, f) = 10 \cdot \log_{10}\left(|\text{Morlet}(t, f) * x(t)|^2\right). \tag{2}$$

For statistical verification, beta-band (15–30 Hz) power segments were extracted and assessed using the Mann-Whitney U test.

For the closed-loop Cyborg-Swarm experiments, we implemented a lightweight online detection algorithm optimized for computational efficiency and artifact robustness. The real-time system processed neural data in sliding windows of 300 samples (0.6 s) with a step size of 15 samples (0.03 s). Raw LFP signals underwent a 50 Hz IIR notch filter and common average referencing. To prevent false triggering caused by motion artifacts, the system monitored signal amplitude. Windows containing channels with absolute amplitudes exceeding $1000\ \mu V$ were flagged as artifact-contaminated. If the proportion of valid channels dropped below 70%, the current window was discarded.

For valid windows, the power spectral density was computed using Welch's method. We extracted the average power in the target beta band (P_β, 15–30 Hz) and a high-frequency control band (P_{HF}, 30–200 Hz). A Fear State was declared only when the beta-band power exceeded the activation threshold ($\lambda_{fear} = 800$), while the high-frequency power remained below the noise rejection threshold ($\lambda_{artifact} = 10$):

$$\text{State} = \begin{cases} \text{Fear (Trigger)}, & \text{if } (P_\beta > \lambda_{fear}) \wedge (P_{HF} < \lambda_{artifact}), \\ \text{Safe}, & \text{otherwise.} \end{cases} \tag{3}$$

Detected events were transmitted to the MouseBot control system via MQTT for millisecond-level actuation.

Supplementary Note 3. MouseBot implementation

The robotic vehicle employs a dual-layer hardware architecture consisting of a Raspberry Pi and an STM32 control board. The Raspberry Pi serves as the upper-level computational unit running the Robot Operating System, simultaneously receiving control commands from an MQTT server via Wi-Fi while acquiring real-time pose data for both the vehicle and the mouse from a motion capture system. The STM32 control board receives PID-based velocity commands from the Raspberry Pi through a serial interface and drives differential motors to achieve precise motion control. During standard task phases, the vehicle operates in a PID algorithm-based following mode, maintaining a predetermined relative position to the mouse in real time.

Pose acquisition is accomplished using a motion capture system that tracks both the vehicle and the mouse. To ensure tracking accuracy, four reflective markers are mounted on the vehicle's top surface, while an additional four markers are attached to a lightweight head-mounted fixture on the mouse. The motion capture system streams pose data for both entities to the ROS-enabled Raspberry Pi via the VRPN protocol.

The vehicle implements a differential drive control strategy governed by

$$v = -k_x e_x, \quad \omega = k_\theta \theta_e, \tag{4}$$

where e_x represents the translational error between the vehicle and the mouse, θ_e denotes the heading angle error, and k_x and k_θ are proportional gain coefficients. An intervention mechanism is triggered upon detection of fear signals from the mouse. The MQTT server transmits an intervention command to the Raspberry Pi, which subsequently sends servo control instructions to the STM32 controller through the serial interface. The STM32 drives the servo motor to rotate, activating a mechanical mechanism that retrieves the mouse into the vehicle compartment. An integrated camera monitors the interior space, with the system determining retrieval status by calculating the pixel proportion of a designated black area. Successful retrieval is confirmed when this black area exceeds 70% of the total pixel count.

Supplementary Note 4. System engineering workflow

The entire experimental system consists of six key components: the MouseBot, adversarial and friendly MAV platforms, a signal processing server, a motion capture system, a communication hub, and the experimental arena. These components operate synergistically to support the closed-loop neural-robot-game experiments.

The MouseBot is directly driven by a flexible brain-machine interface, which maps the mouse's local field potential signals to wheeled motion in real time. The adversarial and friendly MAV platforms introduce threat and assistance roles, respectively, creating a dynamic scenario involving both game and cooperation during gameplay. Each MAV weighs 0.46 kg with a motor-to-motor distance of 0.149 m and a thrust-to-weight ratio of 4.1. Onboard sensors

sample voltage signals at 1000 Hz, while the Vicon system provides 200 Hz pose data.

A high-performance laptop computer serves as the signal processing server, handling parallel signal processing tasks. The platform uses MATLAB for real-time LFP decoding and extraction of key neural patterns. The motion capture system comprises six Vicon cameras mounted on tripods, tracking three-dimensional position, orientation and velocity of the MouseBot and MAVs at 200 Hz. A dedicated computer acts as the communication hub, implementing the lightweight MQTT protocol to coordinate global control across robotic agents.

Supplementary Note 5. Implementation for policy training of MADDPG

We extended the open-source Multi-Agent Particle Environment to design a 2-vs-1 circular pursuit-evasion game. The system consists of three types of agents: a MouseBot, an ally MAV and an enemy MAV. The enemy drone follows a first-order integrator kinematic model:

$$\dot{p}_e = u_e, \quad u_e \in \mathbb{R}^2, \tag{5}$$

while the hybrid robot obeys a non-holonomic differential drive kinematic model:

$$\dot{x}_r = v\cos\theta, \quad \dot{y}_r = v\sin\theta, \quad \dot{\theta}_r = \omega. \tag{6}$$

To induce cooperative interception and adversarial evasion, we design asymmetric reward functions. The total rewards for the MouseBot (r_v), ally (r_{ally}) and enemy (r_{enemy}) are:

$$r_v = r_{\text{pos}} - r_{\text{col}} - r_{\text{ve}} + r_{\text{heading}}, \quad r_{\text{ally}} = r_v - r_{\text{col}} + r_{\text{ae}}, \quad r_{\text{enemy}} = r'_{\text{ve}} - r'_{\text{pos}} - r_{\text{col}} - r_{\text{ae}}. \tag{7}$$

The MouseBot employs a dense positional reward $r_{\text{pos}} = 5.0/(\|\mathbf{p}_m - \mathbf{g}\|_2 + 0.05)$. The enemy's target-distance term $r'_{\text{pos}} = 2.0\|\mathbf{p}_m - \mathbf{g}\|_2^2$ is subtracted to incentivize maximizing the MouseBot's distance from the target. The MouseBot incurs a safety penalty $r_{\text{ve}} = 10\exp(-5d_{me}) + 50\mathbb{I}(d_{me} < 0.5)$, while the enemy receives $r'_{\text{ve}} = 2.0(D^2_{\text{max}} - d^2_{me})$ to encourage interception. The heading reward $r_{\text{heading}} = 2.0(\mathbf{v}_m \cdot \mathbf{u}_g)$ rewards the MouseBot for aligning its heading with the target, and $r_{\text{ae}} = 0.5(D^2_{\text{max}} - d^2_{ae})$ drives the ally to actively pursue the enemy. A uniform penalty $r_{\text{col}} = 5$ is applied for boundary violations.

All agents employ two-layer fully connected actor-critic networks (64 units per layer) trained under the Multi-Agent Deep Deterministic Policy Gradient algorithm following centralized training and decentralized execution. Training was conducted for 150,000 episodes with replay buffer size 10^4, batch size 1024, learning rates of 1×10^{-3} for the actor and 5×10^{-3} for the critic, and discount factor $\gamma = 0.98$. Policy evaluation was performed every 100 episodes.

Algorithm 1 Multi-Agent Training under CTDE

Initialize actor networks $\mu_i(s_i; \theta_i^{\mu})$ and critic networks $Q_i(s, a; \theta_i^{Q})$ for each agent i
Initialize target networks μ'_i, Q'_i and replay buffer $\mathcal{D}$
for each episode **do**
    for each step t **do**
        Agents select actions $a_i = \mu_i(s_i) + \mathcal{N}_t$ and interact with the environment
        Store transition (s, a, r, s') into $\mathcal{D}$
        if training step mod $K = 1$ **then**
            Sample a minibatch from $\mathcal{D}$
            Update critics by minimizing the TD error
            Update actors using the policy gradient
            Soft-update target networks
        end if
    end for
end for
